# Neurobiology of neuropsychiatric symptoms in Alzheimer's disease: A critical review with a focus on neuroimaging

**DOI:** 10.1590/S1980-57642013DN70300002

**Published:** 2013

**Authors:** Lyssandra dos Santos Tascone, Cássio Machado de Campos Bottino

**Affiliations:** 1 Old Age Research Group, Institute of Psychiatry, University of São Paulo, São Paulo SP, Brazil.

**Keywords:** dementia, Alzheimer's disease, neuroimaging, neuropsychiatric symptoms, BPSD

## Abstract

The objective of this critical review of the literature was to reveal the neural circuits involved in the occurrence of neuropsychiatric symptoms (NPS) in Alzheimer's disease (AD) patients through the association of these symptoms with neuroimaging findings. The search for articles was performed on PUBMED from January 2000 to May 2013, using the key words: Dementia AND BPSD; Dementia AND Neuropsychiatric Symptoms; and Dementia AND Psychosis, Delusions, Hallucinations, Agitation, Depression, Anxiety, Apathy, Euphoria, Disinhibition, Irritability, Aberrant Motor Behavior, Sleep or Eating Disorders. Forty-six articles were reviewed and important contributions, especially regarding the psychopathological concepts discussed, were also considered even if not included in this time period. The available evidence suggests the three most relevant neurobiological models for neuropsychiatric symptoms in Alzheimer's disease are the frontal-subcortical circuits, the cortico-cortical networks, and the monoaminergic system. We discussed the association of the individual symptoms or syndromes with these models.

## Abstract (pt)

O objetivo dessa revisão crítica da literatura é investigar os circuitos neurais envolvidos na ocorrência dos sintomas neuropsiquiátricos nos pacientes com doença de Alzheimer através da associação destes sintomas com achados de neuroimagem. A procura dos artigos foi feita no PUBMED de Janeiro de 2000 a Maio de 2013, usando as palavraschave: Demência E BPSD; Demência E Sintomas Neuropsiquiátricos; e Demência E Psicose, Delírios, Alucinações, Agitação, Depressão, Anxiedade, Apatia, Euforia, Desinibição, Irritabilidade, Comportamento Motor Aberrante, Distúrbios do Sono ou Apetite. Quarenta e seis artigos foram revisados e contribuições importantes, especialmente considerando os conceitos psicopatológicos discutidos, foram também discutidos, mesmo se não incluídos neste periodo de tempo. As evidências disponíveis sugerem que os três modelos neurobiológicos mais relevantes para os sintomas neuropsiquiátricos na doença de Alzheimer são os circuitos frontal-subcorticais, as redes cortico-corticais, e o sistema monoaminérgico. Nós discutimos a associação dos sintomas individuais ou síndromes com esses modelos.

## INTRODUCTION

The search for a better understanding and management of the BPSD or neuropsychiatric symptoms (NPS) in dementia includes unveiling the biological mechanisms associated with the emergence of these psychopathological and behavioral manifestations.

The occurrence of NPS in patients with Alzheimer's disease (AD) can be explained in 3 ways: [1] NPS represent an epiphenomenon of AD, or may be the result of the neurodegenerative process in AD; [2] NPS represent biologically different subtypes of AD, such as AD + psychosis or AD + mood disorder; [3] after a certain period of degeneration, genetic factors may be triggered and assume significance in the picture of atrophy of the AD brain.^1^

There are no conclusive evidence about which relationship predominates, but several authors have recognized that if the NPS dementia disorders originate from specific brain systems this would be, in some instances, independent of cognitive impairment.^2-5^

Independence between cognition and behavior was found in factor analysis in patients with AD, suggesting that different neurobiological systems may be implicated in the pathogenesis of these two dimensions and possibly different treatment approach should be used.^3^

The presence of some clinical features of the NPS also supports the notion of independence between NPS and cognition. The features which suggest etiological independence between NPS and cognition are: [1] not all patients with dementia exhibit NPS and NPS occur when the severity and duration are relatively different for each subject; [2] there is a discrepancy between the occurrence of NPS and linear decline in cognitive impairment.^2^

Additionally, other authors have reported negative findings regarding the association between NPS and medial temporal degeneration, suggesting that degeneration of the hippocampus and amygdala cannot be closely correlated with the emergence of NPS in AD as with cognitive decline.^6-8^

In a spectroscopy study, which sought to clarify the pathophysiology of NPSs in AD, the authors found the Mini-Mental State Examination (MMSE), "Clock Drawing Test" (CDT) and "Story Recall Test" (SRT) to be positively correlated with NAA / Cr ratio and negatively correlated with mI / Cr ratio in the posterior cingulate gyrus, but not in the anterior cingulate gyrus. On the other hand, the scores obtained in two categories of the BEHAVE-AD scale – delusional disorders and activity – were negatively correlated with NAA / Cr and positively correlated with mI / Cr in the anterior cingulate gyrus, yet not in the posterior cingulate gyrus. From these findings, the authors concluded that the NPS and decline in cognitive function in AD patients might have different pathophysiologies.^9^

Although findings suggest independence between the global cognitive function and behavioral symptoms, undoubtedly specific cognitive impairments may contribute to the onset or severity of specific NPS.^1^ This hypothesis has been supported by advances in neuroscience that have established the presence of extensive and reciprocal neuronal connection between the epicenters of emotion and cognition.^10^ This theme, however, has not yet been extensively explored in the literature.

In the classic study of neuroanatomy, behavioral symptoms result from lesions in different, and sometimes distal, regions. This occurs because the regulation of many types of behavior and visceral activities do not depend on isolated proximal areas but rely on circuits that may involve very distal areas.^10^

In the present review of the literature, the objective was to present and discuss articles exploring the association of NPS in AD with neuroimaging findings. However, before understanding the neural circuits involved in their manifestations, it seems to us crucial to first explore the neuropsychiatric symptoms, when possible within a unitary concept, where it would be more conducive to the understanding of the neurobiological phenomenon.

The search for articles was performed on the PUBMED database, published from January 2000 to May 2013, using the keywords: Dementia AND BPSD; Dementia AND Neuropsychiatric Symptoms; and Dementia AND Psychosis, Delusions, Hallucinations, Agitation, Depression, Anxiety, Apathy, Euphoria, Disinhibition, Irritability, Aberrant Motor Behavior, Sleep or Eating Disorders. Forty-six articles were reviewed and important contributions, especially regarding the psychopathological concepts discussed, were also considered even if not included in this time period.

In the beginning of each section, we present a brief description of the psychopathological and/or behavioral phenomena assessed in most articles with the NPI scale and their proposed neurobiological hypotheses. The following NPS items or domains were discussed: [1] Psychosis (including delusions and hallucinations); [2] Agitation; [3] Depression; [4] Anxiety; [5] Apathy; and [6] Euphoria, Disinhibition, Irritability, Aberrant Motor Behavior, Sleep and Eating Disorders.

## DISCUSSION

**Psychosis.** Psychosis in patients with AD includes the occurrence of delusions and hallucinations, but delusions occur more frequently than hallucinations.^1,11^

The phenomenological characteristics of delusions in AD comprise two major groups: persecutory delusions and misidentification phenomena. The persecutory delusions are related to ideas of theft, loss, infidelity or abandonment.^11-14^ The misidentification phenomenon was initially described as a shift in perception,^12^ but has been considered in the literature as a type of delusion.^14,15^ Misidentification phenomenology is related to different concepts such as "phantom boarder" (real or imaginary person is living in your home), "mirror sign" (inability to recognize oneself in the mirror), "TV sign" and "picture sign" (inability to differentiate between TV and picture from reality), "Capgras" (caregiver has been replaced by an imposter), "not my house" (inability to recognize one's own home) and "deceased alive" (search for deceased relatives).^11,13^

Hallucinations, on the other hand, can be auditory or visual, but in most cases of AD are visual^1^.

The NPI scale, used in the majority of studies on dementia, involves both persecutory delusions and misidentification syndrome in the characterization of delusions.

Psychosis, therefore, is a complex behavioral disorder that is probably not manifested due to a single brain defect. Changes in reality testing and abnormal inferential thinking changes are also essential for psychosis. This disorder involves deficits in the internal monitoring and executive process in abnormal understanding of relevance of emotional stimuli.^16^

The executive systems and self-monitoring networks of the anterior cingulate cortex (ACC) simultaneously operate specific sensory and associational networks, such as dorsolateral frontal and posterior parietal attentional network, and the connection with subcortical nuclei such as the striatum. The cortical-subcortical circuit between ACC and striatum is responsible for coordinating the integration of emotional tone with the relevant executive processing. Dysfunction of this circuit could generate emotionally changed aberrant beliefs. In addition, dysfunction of the dorsolateral and medial frontal cortical network is associated with the occurrence of psychosis in AD.^16^ The frontal lobe processes that are relevant to episodic memory were also strongly correlated with delusional memories and confabulations in the elderly.^17^

Moreover, the co-activation often observed between ACC and insula, through a variety of cognitive tasks, suggests a functional network involving these two regions. Both functional and structural connectivity has been established between the insula and ACC, probably extending into the inferior frontal region.^18^ The insula, in turn, can be histologically and functionally divided into anterior and posterior portions. The anterior insula is involved in interoceptive stimuli processing, constituting a multisensory integrative high-level process. This integration of different qualities of our experience of the world creates the context for thoughts and actions.^19^ The posterior insula, on the other hand, also connected to the anterior insula, receives an interoceptive representation of the information, being crucial for the experience of self-awareness. Thus, the right posterior insula exerts a key role in the accumulation and release of instantaneous interoceptive information resulting in the perception of complex constructs such as the passage of time. Additionally, functional connectivity has been demonstrated between the posterior insula and medial posterior cingulate region.^20^ The medial posterior cingulate cortex, along with other cortical midline structures, play a crucial role in self-referential processing that favors the construction of the notion of self,^21^ consequently it could contribute to the formation of delusions.

Temporal structures also exert an important role in the genesis of psychotic symptoms. The misidentification syndromes are related to changes in frontal areas,^22^ and are also related to the involvement of the medial temporal lobe.^22,23^ The lateral temporal lobe pathology, on the other hand, especially the superior temporal cortex which controls the representation of the behavior of others, could associate an everyday behavior to something threatening or emotionally significant.^24^ Thus, the superior temporal lobe pathology is related to disorders of thought, but it is important to remember its role in auditory processing and language related to the occurrence of auditory hallucinations.^10^

Secondary visual processing centers such as the lateral temporal and occipital-parietal areas act in misidentification syndromes.^24^ The presence of auditory and visual hallucinations in temporal limbic epilepsy also contributes to the understanding of the neural hallucinations, where changes in temporal lobe with extension to limbic structures contribute to the onset of this psychopathological phenomenon.^10^

The presence of distortion of reality in psychosis is associated with functional alterations in lateral prefrontal cortex, striatum, in the superior temporal gyrus and parahippocampal region.^25^

From these data, we can summarize that, in AD patients, the brain areas involved in the pathogenesis of psychotic symptoms are the anterior cingulate cortex, the insula, and regions in the frontal, temporal, and parietal lobes, depending on which symptoms or syndromes arise.

**Agitation.** Agitation can be defined as an inappropriate verbal, vocal or motor activity that results directly from the individual's needs or from a confusional state of the individual who manifests it.^1^

There are different characterizations of agitation. The Cohen-Mansfield Agitation scale, for example, classifies nonaggressive physically, non-verbally aggressive, physically aggressive or verbally aggressive behaviors as agitation.^26^ The NPI scale emphasizes the resistance to physical and verbal care.^14^ AD patients are often difficult to handle, becoming physically and verbally aggressive with their caregivers.

The uniformity in the definition of agitation in AD studies is essential to facilitate the comparison of results and the proper investigation of the neurobiological aspects associated with the clinical phenomenon. Depending on the study, agitation can refer to motor disorders including wandering, restlessness and other disorganized behavior, while the concept is also used to refer to verbal or physical abuse.^27^

Moreover, evidence from human studies suggests that the prefrontal cortex, hippocampus and ACC are the major components of the regulatory circuitry of aggression.^27^ Cortical areas such as the dorsolateral prefrontal cortex (DLPFC) and the orbitofrontal cortex (OFC) have been associated with emotions. The DLPFC and OFC receive input from sensory and limbic amygdala and other medial temporal areas and integrate this information. Damage to these areas has an impact on processing of emotional behavior, resulting in problems with emotion regulation and subsequent difficulties with inhibitory and aggressive behavior.^28^

In AD, agitation is associated with executive dysfunction and more severe functional impairment,^1^ developing as a result of the interaction of neurobiological and environmental factors.^29^ Neuropathological studies have found a correlation between higher scores of agitation and deposits of neurofibrillary tangles in bilateral OFC regions and ACC.^29^ Agitation was shown to be correlated with hypometabolism in frontal and temporal lobes in AD patients.^29,30^ Additionally, a functional study demonstrated temporal limbic cortical hypoperfusion was linked with aggression.^31^

In summary, these results suggest that the frontal region, the ACC and limbic areas are involved in the pathophysiology of agitation/aggression in AD.

**Depression.** Depression involves a number of emotional symptoms such as crying, sadness, anxiety, feelings of hopelessness, capital loss, and recurrent thoughts of death. Additionally, the picture is characterized by the presence not only of emotional symptoms, but also behavioral, cognitive and somatic symptoms. The traditional diagnosis of depression (DSM-IV) also includes one aspect of apathy.^32^ But this construct requires a different clinical approach in AD cases in which the assessment of depression and apathy must be performed separately.^15,32^ In a comparison study of NPS in 5 different neurodegenerative disorders, including AD, the authors found no correlation between depression and apathy in 154 patients and concluded that the diagnosis of apathy should be separated from depression.^32^

In the neurobiology of depression, the neural networks that regulate the aspects of normal emotional behavior have also been implicated in the pathophysiology of affective disorders. The prefrontal cortex, limbic structures and related pallidal-striatal-thalamic structures organize emotional expression. Thus, the three circuits most relevant to depression are those incorporating regions of the OFC, ACC^33,34^ and DLPFC.^33-35^ Orbitofrontal dysfunction could produce characteristic states of depression with reduced ability to interrupt the perseverative melancholic thoughts and the responses to ordinarily nonthreatening anxious stimuli. The anterior cingulate cortex was involved in motivational and emotional information assessment and the regulation of emotional response in depression.^36^

The prefrontal cortex, particularly the dorsolateral and ventromedial sectors, has been the focus of increased attention in relation to their involvement in the pathogenesis of depressive symptoms.^35^ Functional neuroimaging data suggests that an imbalance in the activity of the ventro-medial PFC (VMPFC) and the dorso-lateral PFC (DLPFC) may contribute to depression, with each of these structures having seemingly opposite activity profiles. The VMPFC is hyperactive at rest and decreases in activity during remission of depressive symptoms, while the DLPFC is hypoactive at rest and increases in activity during remission.^35^

Consequently, the DLPFC and VMPFC apparently mediate different neurocognitive / neurobehavioral mechanisms. The VMPFC performs a basic function in the generation of negative emotion, and its activity is correlated with subjective experience of negative affect. An alternative possibility is that the VMPFC is related to self-awareness and self-reflection thus contributing to certain negative emotions such as guilt, shame, embarrassment and regret.^35^ The DLPFC, on the other hand, has been associated with executive functions such as manipulation and control of items in working memory, intention formation, goal-directed action, abstract thinking and attention control. In addition, studies suggest that cognitive control functions mediated by DLPFC may also participate in emotion processing. Specifically, functional studies demonstrate the recruitment of the DLPFC during regulation of negative emotion through strategic reappraisal / suppression. If the revaluation / suppression of negative affect is a protective mechanism against depression, and DLPFC is the key neural substrate for this function, then the DLPFC plays a critical role in depression. Thus, a dysfunction in the regulation of negative affects due to DLPFC hypofunction would be a plausible mechanism for the involvement of this structure in depression.^35^

The portions of the prefrontal cortex (PFC) involved in depression are part of a larger system called the "default system" which include the dorsal portion of the PFC, the middle and posterior cingulate cortex, the anterior temporal cortex, and parahippocampal and entorhinal cortex, allegedly involved in the functions of self-reference. This system has no substantial sensory connections but has prominent connections with the limbic system and visceral control structures (hypothalamus and periaqueductal gray).^33^ This system, called the visceromotor system, is involved in functions including introspective mood and emotion, and visceral reactions to emotional stimuli.^33^ The PFC and its related limbic structures, through the modulation of visceral control structures, may explain the disturbances of autonomic regulation and neuroendocrine responses that are associated with mood disorders.^33^

In addition to the involvement of cortical-limbic circuitry described, the limbic-cortical-striatal-thalamicpallidum circuit, which supports the involvement of basal ganglia in depression, is associated not only with the pathophysiology of early-onset depression, but with the late onset depression.^36^ VBM studies in early-onset depression have shown reduced gray matter in the lateral frontal lobe, anterior cingulate cortex, lateral and medial temporal lobe, amygdala-hippocampus complex,^37,38^ and insular cortex.^38^ In late-onset depression, studies show hippocampal,^36,39,40^ DLPFC,^41^ orbitofrontal cortex,^36,42^ temporal lobe,^36,42^ bilateral precuneus and posterior cingulate cortex atrophy.^43^ The findings of functional neuroimaging in AD also support the hypothesis of involvement of these structures in depression, since depression was associated with hypoperfusion measured by PET in the anterior cingulate, prefrontal cortex,^31,44^ upper and middle temporal cortices.^44^

**Anxiety.** The most common clinical form of anxiety in dementia is generalized anxiety disorder (GAD). Other symptoms include Godot syndrome in which the patient repeatedly asks questions related to an upcoming event, exhibit fear of being alone and wandering behaviors, rubbing of hands, fidgeting and humming.^1,45^ Few patients with AD and anxiety symptoms show all the diagnostic criteria for GAD in DSM.^1,46,47^ Thus, criteria for the differential diagnosis of anxiety in AD would be useful. To this end, the diagnostic construct of anxiety in AD was validated in a study that evaluated 554 patients with probable AD. The criteria included symptoms of restlessness, irritability, muscle tension, fears, and respiratory symptoms in the context of excessive anxiety and worry.^47^

With regard to how anxiety arises in the context of AD, several authors agree that anxiety is often associated with other neuropsychiatric symptoms such as depression, psychosis, aberrant motor behavior, disinhibition, euphoria, irritability, and agitation.^1,45,46^

In view of the scant evidence regarding the neural mechanisms involved in AD anxiety, the neurobiological mechanisms of anxiety in healthy subjects and generalized anxiety disorder in adults could contribute toward better understanding this phenomenon in AD. Structural changes in the medial orbitofrontal cortex and ACC seem to be related to the anxiety trait.^48^

The ACC has been implicated in the mechanism of fear extinction and emotion regulation. In addition, the ACC acts as a regulatory point between the DLPFC and amygdala, modulating the responsiveness of the amygdala.^37^ Consequently, the amygdala, the ACC and prefrontal cortex play a role in processing of fear in anxiety.^48^

Concerning results of studies on Generalized Anxiety Disorder in adults, the presence of abnormalities in regions responsible for emotional processing and emotional behaviors such as the amygdala, prefrontal cortex and temporal lobe were found. There are structural neuroimaging findings that support this hypothesis, presenting results of anatomical abnormalities in the amygdala and temporal lobe, specifically the superior and middle temporal gyrus, in association with the diagnosis of Generalized Anxiety Disorder, and ACC.^37^

In a PET study, which sought to determine the relationship between anxiety and brain metabolism in 41 patients with mild-moderate AD patients, the authors reported that higher anxiety scores in NPI were correlated with hypometabolism in the bilateral entorhinal cortex, anterior parahippocampal gyrus, superior temporal gyrus and left insula, unrelated to cognition.^45^

Currently, there is a lack of studies investigating the brain areas associated to anxiety in AD patients. Based on the results of studies in adults and a functional study with AD patients, anxiety is likely associated with changes in frontal, lateral and medial temporal lobe, insula and the ACC.

**Apathy.** Apathy is defined as diminished motivation in affect, behavior and cognition, leading to a loss of responsiveness to stimuli evidenced by loss of self-initiated behaviors, not attributable to decreased level of consciousness, cognitive impairment, or emotional distress.^32,49^ Its clinical manifestations are loss of interest, motivation, volition, involvement, spontaneity and emotional behavior.^50^

Clinical signs of apathy are a common feature of injury or dysfunction in the prefrontal cortex and basal ganglia, one of the functional systems involved in the generation and control of self-generated goal-directed behavior.^51^ Of these structures, the anterior cingulate cortex seems to be a brain structure critical for the initiative, motivation, expression of affection and goal-directed behavior.^52^

Considering the findings of functional studies and experimental data involving ventromedial PFC in decision-making, a mechanism for reduced activation in goal-directed behaviors was proposed to explain the occurrence of apathy in AD. This mechanism is initiated by a dysfunction in the evaluation of the action, which is executed by the interaction of the prefrontal cortex, amygdala and ACC. This deficit compromises the proper transmission of the signal to the nucleus accumbens, determining the non-activation of dopaminergic midbrain ascending pathways, areas needed to engage the dorsal striatum. Consequently, the striatum becomes deficient in extracting the most appropriate response to be executed from the frontostriatal circuit.^53^

Additionally, structural neuroimaging studies report an association of apathy in AD with decreased gray matter volume in areas mentioned above such as the ACC bilaterally, frontal cortex bilaterally,^1,4,50^ putamen and head of the left caudate nucleus.^4^

Data reviewed suggest that cortical structures involved in the pathophysiology of apathy in AD include the frontal cortex (prefrontal and orbitofrontal), anterior cingulate cortex, amygdala and basal ganglia.

**Euphoria, disinhibition, irritability, aberrant motor behavior, sleep and eating disorders.** The research on the neurobiology of these symptoms in AD is limited, probably because they are less frequent than the others,^1^ often associated with other NPS, or fail to show correlation with the neurobiological construct defined.

Disinhibition could be correlated with prefrontal cortex involvement, especially its ventromedial portion. Studies of brain lesions associated VMPFC damage with loss of self-insight, as well as a reducing negative affect, particularly shame, guilt, embarrassment and regret.^35^ There is evidence of the association of reduced gray matter with bilateral disinhibition in ACC and right middle frontal gyrus in AD^23^ and decreased subgenual cingulate gyrus in FTD / semantic dementia.^54^ Furthermore, personality changes and emotional lability with disinhibition in dementia are evident in orbitofrontal-subcortical circuit dysfunction of the cortical portion.^55^

The irritability symptom has often been associated with other symptoms generating syndromes with different characteristics. It appears in hyperactivity syndrome with disinhibition, aberrant motor behavior,^3^ agitation^56,57^ and euphoria^57^. However, irritability is also associated with the syndrome of psychosis and affective symptoms such as depression.^58^ Moreover, VBM studies have shown no association between this symptom and structural changes in AD or other dementias.^4,23,54,59^ This evidence indicates this is a symptom without specific neurobiological substrates, which probably occurs overlapping with the neural correlates of other neuropsychiatric symptoms.

Regarding the presence of aberrant motor behavior, one study investigating the association between frontal lobe function and activity disturbances (wandering, aimless and inappropriate activities) showed that patients with AD and these behavioral symptoms had significantly lower performance on subtests of frontal functions (inhibitory control and verbal fluency). Verbal fluency tasks also require the skill of well-organized retrieval from semantic memory and flexible behavioral adaptation to new situations. The aberrant motor behavior, including wandering or inappropriate activity, could be caused by an inability to react flexibly after a stimulus variable from the environment. Among patients with AD, in addition to changes in memory and visual-spatial deficits, loss of self-regulation executive functions could cause deficits in the ability to perform tasks efficiently, leading to wandering and inappropriate or purposeless activity.^60^ Thus, one would expect to find structural changes in the frontal lobe.

Sleep disorders are common in AD and other dementias. Disturbance of the sleep-wake cycle is the most prominent sleep disorder in AD.^61^ The sleep disorders in AD are associated with damage to the suprachiasmatic nucleus, although some of the pathogenesis of these disorders is known.^62^

In relation to eating disorders, decreased appetite is more common in AD.^63^ Thus, it is likely to occur in AD subsequent to involvement of structures of motivational control of appetite. Motivational control of appetite in humans is influenced by intrinsic and extrinsic motivation. Intrinsic factors involve structures of the anterior cingulate cortex and its connected structures (amygdala, ventral striatum, hypothalamus, insula, and orbitofrontal cortex), such as the circuit of visceromotor and endocrine associated functions activated in response to starvation. The neural substrates of extrinsic factors comprise the amygdala and orbitofrontal cortex involved in the processing of the stimulus to promote appetite and subsequent production of goal-directed behavior of finding and selecting a desirable food.^64^ Additionally, studies show functional activation of ACC during a hunger state, volitional swallowing, as well as gustatory and olfactory processing. Thus, atrophy of brain regions intimately involved in food might contribute to altered signals in appetite which in turn contribute to the resulting weight loss seen in AD.^65^

For these individual symptoms, on which there is limited evidence of their neurobiological substrates, we can confirm the involvement of the frontal cortex, anterior cingulate cortex and insula in the pathophysiological process of NPS.^4,23,54,59^

In conclusion, the available evidence suggests that the three most relevant neurobiological models for neuropsychiatric symptoms in Alzheimer's disease are the frontal-subcortical circuits, the cortico-cortical networks, and the monoaminergic system. The most relevant frontal-subcortical circuits in AD are the dorsolateral prefrontal, orbitofrontal, and anterior cingulate, circuits dedicated to executive functions, social behavior, and motivational states in humans, respectively. Each circuit has a frontal component, a substrate of the basal ganglia and a thalamic component, returning to its original frontal connection. Regarding corticocortical networks, one of these is the memory-emotion network whose epicenters are the hippocampus and the amygdala, which have extensive reciprocal connections conferring an intimate relationship between the processes of memory and emotion. The general organization of the monoaminergic system consists of cell bodies of neurons producing serotonin, norepinephrine or dopamine. These neurons located in the brain diffusely project long axons to virtually all parts of the brain to mediate human behavior.

A better understanding of the neural correlates of each neuropsychiatric symptom and their related syndromes in AD patients may help improve the management of these problems in the future, leading to more specific medications and efficient non-pharmacological strategies for treatment.
